# Mobility Patterns and Mental Health During the COVID-19 Pandemic: Longitudinal Observational Study Using Smartphone Mobility Data

**DOI:** 10.2196/79711

**Published:** 2026-07-27

**Authors:** Owen Ngo Wang Leung, Linda Chiu Wa Lam, Ronald C Kessler, Pim Cuijpers, Samuel Yeung Shan Wong, Arthur Dun Ping Mak

**Affiliations:** 1 Department of Psychiatry Faculty of Medicine Chinese University of Hong Kong Hong Kong SAR China (Hong Kong); 2 Department of Health Care Policy Harvard Medical School Boston, MA United States; 3 Department of Clinical, Neuro and Developmental Psychology Vrije Universiteit Amsterdam Amsterdam The Netherlands; 4 Jockey Club School of Public Health and Primary Care Faculty of Medicine Chinese University of Hong Kong Hong Kong SAR China (Hong Kong)

**Keywords:** digital mental health, mobility patterns, COVID-19, pandemic, smartphone data, digital phenotyping, longitudinal analysis

## Abstract

**Background:**

The COVID-19 pandemic disrupted mobility globally, but its mental health implications remain difficult to characterize because most studies relied on lockdown status, population-level mobility indicators, or self-reported mobility. These approaches may miss individual differences in actual movement patterns and cannot fully examine bidirectional relationships between mobility and mental health. Individual-level smartphone geolocation data may provide a more objective and temporally aligned measure of mobility during periods of societal disruption.

**Objective:**

This study aimed to use individual-level Google location history (GLH) data and population-level Google community mobility reports (GCMRs) to examine concurrent and longitudinal relationships between pandemic-era mobility patterns and mental health symptoms in Hong Kong.

**Methods:**

This study analyzed data from the CU-COVID19 cohort study, an online longitudinal survey study of the psychological impact of the pandemic in Hong Kong. Mental health symptoms over the previous 14 days were assessed at baseline, 6 months, and 12 months using the 9-item Patient Health Questionnaire, the 7-item Generalized Anxiety Disorder scale, and the 4-item PTSD Checklist for DSM-5. Participants provided retrospective GLH data reflecting their mobility during the corresponding 14-day survey periods. The analytic sample included 145 participants with baseline GLH data, of whom 110 had 6-month follow-up data and 49 had data available at all 3 assessment waves. GLH data were used to derive mobility factors representing journey diversity, immobility, and remoteness. Population-level mobility during the same 14-day periods was measured using Hong Kong GCMR residential stay data. Concurrent mediation models examined whether individual mobility mediated associations between population-level residential stay and mental health symptoms. Longitudinal models examined bidirectional associations between changes in individual mobility and mental health across 6-month intervals.

**Results:**

Population-level residential stay was not directly associated with mental health. In concurrent mediation models, higher population-level residential stay was associated with lower individual journey diversity (β=–0.36; *P*<.001), and lower journey diversity was associated with higher depression (β=–0.29; *P*=.02) and posttraumatic stress disorder (PTSD) (β=–0.35; *P*=.002). Bootstrapped indirect effects suggested mediation through journey diversity for depressive symptoms (β=0.11, 95% CI 0.02-0.25) and PTSD symptoms (β=0.13, 95% CI 0.05-0.27), although the depression-related indirect effect became less robust after adjustment for local and individual COVID-19 infection indicators. Longitudinally, higher baseline depressive symptoms predicted subsequent reductions in journey diversity (β=–0.15; *P*=.02), and reductions in journey diversity predicted higher subsequent depressive symptoms (β=–0.43; *P*=.008).

**Conclusions:**

Individual-level mobility patterns, particularly lower journey diversity, showed more consistent associations with mental health symptoms than population-level residential stay. Findings suggest bidirectional relationships between mobility and mental health and demonstrate the potential of smartphone geolocation data for digital phenotyping. However, the modest and self-selected sample, limited GCMR availability, and observational design require cautious interpretation.

## Introduction

The COVID-19 pandemic saw unprecedented changes in human mobility patterns. Domestic movement declined to 59% of prepandemic levels across 135 countries during initial lockdowns [1]. Fundamental shifts in travel purpose, routes, modes, and timing have occurred since the pandemic and have persisted beyond its conclusion [2]. These mobility transformations warrant public health attention because mobility facilitates access to outdoor activities, an important correlate of mental health [3]. Moreover, the widespread mobility disruptions caused by the pandemic created a unique natural experiment for examining the relationship between mobility changes and mental health, an association that remains incompletely understood.

Previous pandemic studies on mobility and mental health have commonly used regional lockdown status as a proxy measure for mobility restrictions [4,5]. More recent large-scale longitudinal studies have used contextual Google mobility indicators to examine associations between spatial immobility and mental health, but these studies still could not directly observe individual-level mobility patterns [6,7]. This limitation introduces measurement errors by overlooking important individual variations in occupational engagement, lifestyle, and risk perception that would have different impacts on mobility patterns [8] and precludes examination of potentially bidirectional relationships between mobility and mental health trajectories over time.

Self-report measures of individual mobility, while providing longitudinal data [9], are labor intensive, prone to recall biases [10], and often miss nuanced changes (eg, idle time, travel distance, or diversity of travel). Consequently, methodological challenges in accurately measuring mobility have hindered the characterization of the relationship between mobility and mental health.

Mobile geolocation data offer a methodological solution to these limitations by providing objective and continuous measurement of individual mobility patterns. Traditional implementations of mobile geolocation data involved distributing dedicated tracking devices or apps, which are costly, restrict sample size, and cannot capture data before study initiation. Google location history (GLH), an opt-in feature of Google Maps [11], offers a promising solution for retrospective individual mobility tracking. GLH continuously records users’ geocoordinates in their Google accounts, enabling data retrieval from before study initiation—a critical advantage for investigating unforeseen events such as the COVID-19 pandemic.

GLH provides high-quality mobility data, with spatial resolution typically under 200 meters and temporal frequency averaging 4.5 minutes [12,13]. Additionally, Google aggregates individual GLH data into population-level metrics through Google community mobility reports (GCMRs) [14]. This combination of detailed individual-level tracking and population-level data uniquely positions GLH and GCMRs to examine the relationship between pandemic-era mobility changes and mental health at both individual and population levels.

The present study analyzes GLH and GCMR data from 145 participants in the CU-COVID19 cohort study in Hong Kong [15]. Baseline mental health assessments via web-based surveys were collected from 2020 to 2023, followed by 2 additional surveys at 6-month intervals. At each survey, participants reported mental health symptoms experienced during the previous 14 days, while GLH and GCMR mobility data were extracted for these same 14-day periods.

Hong Kong provides a unique context, as the government implemented social distancing rather than lockdowns [16], resulting in mobility changes through both policy and voluntary actions. This created significant population-level mobility change while maintaining individual agency, highlighting the importance of individual-level data.

This study leverages Hong Kong’s unique pandemic response characteristic and novel data sources to address two key research questions: (1) How were mobility changes at both population and individual levels related to mental health? and (2) What bidirectional relationships existed between individual mobility patterns and mental health symptoms over time? Additionally, this research explored the feasibility and utility of GLH for retrospective mobility assessment in health research, addressing a significant methodological challenge in investigations of the relationship between mobility and health.

## Methods

### Participants and Study Design

Participants were recruited from the CU-COVID19 project examining the pandemic’s psychological impact in Hong Kong through online surveys [15]. The cohort study used a rolling recruitment strategy, with baseline surveys completed between November 2020 and October 2023 and 2 follow-ups at 6-month intervals. The survey time points are hereafter referred to as wave 1 (baseline), wave 2, and wave 3.

Participants in the CU-COVID19 study were eligible if they were aged 18 to 75 years, able to read Chinese, and had access to an internet-enabled device. The parent cohort recruited 4 groups: individuals diagnosed with COVID-19 through the Chinese University of Hong Kong–affiliated clinics and social media campaigns, individuals in isolation or quarantine at Penny Bay Quarantine Camp, health care workers from 3 major hospitals in Hong Kong, and the general public through study invitations mailed to a random stratified selection of household addresses obtained from the Census and Statistics Department.

Starting in August 2022, when the GLH substudy was launched, CU-COVID19 participants who were still completing their 6- or 12-month follow-up surveys were systematically invited to provide GLH data (n=2508). Participants who had completed all the follow-up surveys before the GLH substudy began were not approached for GLH data. A total of 1340 participants indicated interest in the GLH component of the study and provided separate electronic consent to receive instructions for checking, extracting, and submitting their GLH data. To minimize response bias, all consenting participants were contacted up to 3 times via phone or messenger to support GLH data extraction and submission. Of these, 310 participants completed the GLH data-checking process, of whom 145 provided GLH data for at least 7 days of the 14-day baseline survey measurement period and were included in the GLH analytic sample. Of the 145 participants, 110 also had GLH data for wave 2, and 49 had data for all 3 time points. A recruitment flowchart is presented in Multimedia Appendix 1.

At each survey wave, 3 types of data were collected: individual-level reports of mental health over the prior 14 days, individual-level mobility during that same period, and population-level mobility data during that same period.

### Mental Health

Validated Chinese versions of the 9-item Patient Health Questionnaire (PHQ-9) [17], the 7-item Generalized Anxiety Disorder scale (GAD-7) [18], and the 4-item PTSD Checklist for DSM-5 (PCL-5) [19] were used to screen for depression, anxiety, and posttraumatic stress disorder (PTSD) symptoms, respectively. Scores ≥10 on PHQ-9 and GAD-7 and >7 on PCL-5 indicated positive screens for depression, anxiety, and PTSD. Participants also reported their history of mental health service use (counseling or psychiatric medication) and previous mental health conditions. History of mental health service use and mental health conditions were used to describe the sample and were not included as covariates in the regression models.

### Individual Mobility

GLH data consisted of time-stamped geolocation coordinates and processed semantic data of places visited (Multimedia Appendix 2). Semantic data represented stationary periods that Google labeled as the most probable place visited. For each wave, GLH data were preprocessed following the method proposed by Palmius et al [20]: filtering speeds >100 km/h, down-sampling to 5-minute intervals, and classifying locations as stationary or moving using a 1.5 km/h threshold. Preprocessing steps are detailed in Multimedia Appendix 3.

Twenty mobility indexes were derived to capture individual differences in basic mobility levels, travel times, time and distance from home and work, and the types of places visited (definitions are provided in Multimedia Appendix 4). Factor analysis reduced these indexes to three dimensions: (1) *journey diversity*: number of trips, number of unique places, recreational visits, transport hub visits, and places visited per outing; (2) *immobility*: stationary time, home time, workplace time, and inverse outing duration; and (3) *remoteness*: trip duration, distance to locations, and the inverse proportion of travel done on foot.

Longitudinal confirmatory factor analysis validated these factors across the 3 waves (comparative fit index or Tucker-Lewis index >0.95, root mean square error of approximation <0.06, standardized root mean squared error <0.1). Detailed factor analysis results and validation metrics are provided in Multimedia Appendix 5.

### Population Mobility

GCMRs provided daily residential stay data for Hong Kong, expressed as a percentage change from the prepandemic baseline (median values for each day of the week from January to February 2020) [14]. Residential stay is an inverse indicator of population mobility; higher percentages reflect reduced population movement. For each survey response, a personalized 14-day average of daily residential stay values preceding that survey date was calculated, creating a measure of population mobility specific to the time when each participant completed their assessment. This mobility measure was computed if Google provided data for at least 7 days of the 14-day period and was available for 86, 62, and 23 participants at waves 1, 2, and 3, respectively.

### COVID-19 Infection Measures

Individual COVID-19 infection status was assessed using wave-specific survey items, indicating infection history at wave 1 and infection in the previous 6 months at waves 2 and 3. Local infection count was derived from Hong Kong Government COVID-19 surveillance data using the log1p-transformed 14-day mean daily confirmed cases over the 14 days preceding each survey [21]. The 14-day window matched the mental health and mobility assessment period.

### Analysis

#### Concurrent Analysis

Relationships between population mobility, individual mobility, and mental health were analyzed cross-sectionally, combining data from all the waves. Cluster-robust sandwich estimators were used to adjust the SEs to account for within-participant correlation [22].

The analysis investigated concurrent associations: population mobility with individual mobility (path A), individual mobility with mental health (path B), and individual mobility as a mediator between population mobility and mental health (path A×B). These models also estimated the direct association between population mobility and mental health after accounting for the corresponding individual mobility factor. To describe the unadjusted population mobility–mental health association outside the mediation framework, we additionally estimated total associations between population-level residential stay and mental health symptoms without including individual mobility factors.

Exploratory moderation models tested interactions between individual mobility factors and the number of household members. Sensitivity analyses repeated the concurrent mediation models with either local infection count or individual infection status included as an additional covariate.

#### Longitudinal Analysis

Bidirectional temporal relationships between mental health and mobility were investigated using pairs of consecutive waves (baseline to wave 2, and wave 2 to wave 3). In analyzing the relationship between mental health and subsequent mobility, we tested whether initial mental health status or changes in mental health were associated with subsequent mobility outcomes. Conversely, we examined if initial mobility level or changes in mobility predicted subsequent mental health outcomes. Each analysis included baseline controls to isolate temporal change effects. Mediation analyses tested whether associations between consecutive waves of one variable were mediated by changes in the other variable.

#### Weighting, α Level, and Programs

Demographics and the mental health of GLH participants (n=145) were compared to those who were invited but did not provide GLH data (non-GLH participants: n=2363) using 1-tailed *t* tests and chi-square tests. On the basis of identified differences, we applied statistical weighting to demographic variables in our analyses to reduce potential selection bias (Multimedia Appendix 6). All analyses used an α level of .05. Mediation relationships were tested using 10,000 bootstrapped samples, with significance indicated by 95% CIs excluding 0 and both original paths being significant at *P*<.05. All regression coefficients presented are standardized. Factor analysis and concurrent analysis were conducted using Mplus version 8.8 (Muthén and Muthén), while longitudinal analyses were performed using *statsmodels* version 0.14.4, an open-source Python package [23].

### Ethical Considerations

All procedures adhered to the ethical standards outlined in the Declaration of Helsinki and were approved by the Joint Chinese University of Hong Kong-New Territories East Cluster Clinical Research Ethics Committee (2021.207 [GLH study] and 2020.338 [CU-COVID19 project]). Participants provided electronic informed consent before participating in the CU-COVID19 survey study, and participants who joined the GLH substudy provided separate electronic informed consent before receiving instructions for GLH data extraction and submission.

Participant privacy and confidentiality were protected through study identifiers and restricted data access. GLH files downloaded from Google Takeout did not include participants’ names, phone numbers, or email addresses, and submitted GLH files were identified by study ID. GLH data were submitted electronically through secure study channels and stored on a password-encrypted hard disk kept in the research laboratory. Analytic datasets were deidentified before analysis and were accessible only to authorized research personnel.

Participants received supermarket vouchers worth HK $100 (HK $1=US $0.13 as of July 7, 2026) for each survey wave, as well as an additional HK $200 for participating in the GLH substudy.

## Results

### Sample Characteristics

The sample (n=145) comprised adults with a mean age of 46.9 (SD 15.5) years, with balanced gender distribution (n=72, 49.7% female participants and n=73, 50.3% male participants). Most of the participants were married (n=80, 54.9%) and employed (n=96, 66.2%), with 20.7% (n=30) retired. Over half (n=74, 51%) of the participants had completed tertiary education, while 9.7% (n=14) reported monthly household income below HK $14,000. The mean household size was 3.3 (SD 1.4) members, and 9 participants (n=9, 6.2%) lived alone. At baseline, 74 (51%) participants reported prior COVID-19 infection. At baseline, the Hong Kong population spent 7.9% more time at home compared to prepandemic levels (Table 1).

Compared to non–Google location history (GLH) CU-COVID19 participants, GLH participants were older, more often male, and had lower educational attainment. A higher proportion of GLH participants were unemployed and had lower household incomes (see Multimedia Appendix 6 for details).

At baseline, mean severity scores were 4.4 (SD 4.7) for PHQ-9, 3.4 (SD 4.1) for GAD-7, and 2.6 (SD 3.1) for PCL-5, with 12.4% (18/145), 7.6% (11/145), and 12.4% (18/145) screening positive for depression, anxiety, and PTSD, respectively. In total, 24.3% (35/145) reported a history of a mental health disorder, and 12.4% (18/145) had sought mental health treatment previously (Table 1).

Across the 3 waves, mental health symptoms and population-level residential stay patterns remained stable (*P* values >.05). Individual mobility patterns showed changes over time, with journey diversity (*P*<.001) and remoteness (*P*=.002) increasing from baseline to follow-up assessments, while immobility remained stable (Multimedia Appendix 7).

**Table 1:** Sample characteristics and population mobility at baseline (n=145).

Variables				Values
**Demographics**				
	Age (years), mean (SD)			46.9 (15.5)
	**Gender, n (%)**			
		Female	73 (50.3)	
		Male	72 (49.7)	
	**Marital status, n (%)**			
		Single	50 (34.7)	
		Married	79 (54.9)	
		Divorced	9 (6.3)	
		Widowed	6 (4.2)	
	**Education, n (%)**			
		Primary or below	13 (9)	
		Secondary	58 (40)	
		Tertiary or above	74 (51)	
	**Employment, n (%)**			
		Employed	96 (66.2)	
		Housekeeper	5 (3.4)	
		Others	2 (1.4)	
		Retired	30 (20.7)	
		Student	4 (2.8)	
		Unemployed	8 (5.5)	
	**Household income (HK $), n (%)**			
		≤13,999	14 (9.7)	
		14,000-35,999	56 (38.6)	
		36,000-57,999	30 (20.7)	
		≥58,000	45 (31)	
	Household members, mean (SD)			3.3 (1.4)
	Living alone, n (%)			9 (6.2)
**Mental health**				
	**Mental health symptom severity, mean (SD)**			
		PHQ-9^a^	4.4 (4.7)	
		GAD-7^b^	3.4 (4.1)	
		PCL-5^c^	2.6 (3.1)	
	**Mental disorder^d^, n (%)**			
		Depression	18 (12.4)	
		Anxiety	11 (7.6)	
		PTSD^e^	18 (12.4)	
	**Past mental disorder^f^, n (%)**			
		Depression	12 (8.3)	
		Anxiety	23 (16)	
		Any mental disorder	35 (24.3)	
	**Mental health treatment, n (%)**			
		Lifetime	18 (12.4)	
**COVID-19 infection**				
	Prior COVID-19 infection, n (%)			74 (51.0)
**Population mobility**				
	GCMR^g^ home stay, mean (SD)			7.9 (3.5)

^a^PHQ-9: 9-item Patient Health Questionnaire.

^b^GAD-7: 7-item Generalized Anxiety Disorder scale.

^c^PCL-5: PTSD Checklist for DSM-5.

^d^Positive screenings for depression, anxiety, and posttraumatic stress disorder were defined as scores of ≥10 on the PHQ-9 and GAD-7 and a score of ≥7 on the PCL-5, respectively.

^e^PTSD: posttraumatic stress disorder.

^f^Any past disorder or symptom related to depression, mania, or bipolar disorder; panic attacks or panic disorder; anxiety-related problems; problems with alcohol consumption; and abuse, dependence, or problems with drug use.

^g^GCMR: Google community mobility report.

### Concurrent Associations Between Population Mobility, Individual Mobility, and Individual Mental Health

In total effect models without adjustment for individual mobility, population-level residential stay was not significantly associated with PHQ-9 (β=0.17, 95% CI –0.15 to 0.42; *P*=.17), GAD-7 (β=0.16, 95% CI –0.18 to 0.45; *P*=.27), or PCL-5 symptoms (β=0.22, 95% CI –0.10 to 0.50; *P*=.07; Multimedia Appendix 8). In mediation models, the direct effects of population-level residential stay on mental health symptoms also remained nonsignificant after accounting for the corresponding individual mobility factor (Table 2).

However, reduced individual journey diversity was associated with increased population-level residential stay (path A: β=−0.36; *P*<.001) and greater severity of depression (path B: β=−0.29; *P*=.02) and PTSD symptoms (path B: β=−0.35; *P*=.002; Table 2). In exploratory moderation analyses, the number of household members did not significantly moderate associations between individual mobility factors and mental health symptoms (all interaction *P* values >.05; Multimedia Appendix 9).

**Figure 1:**
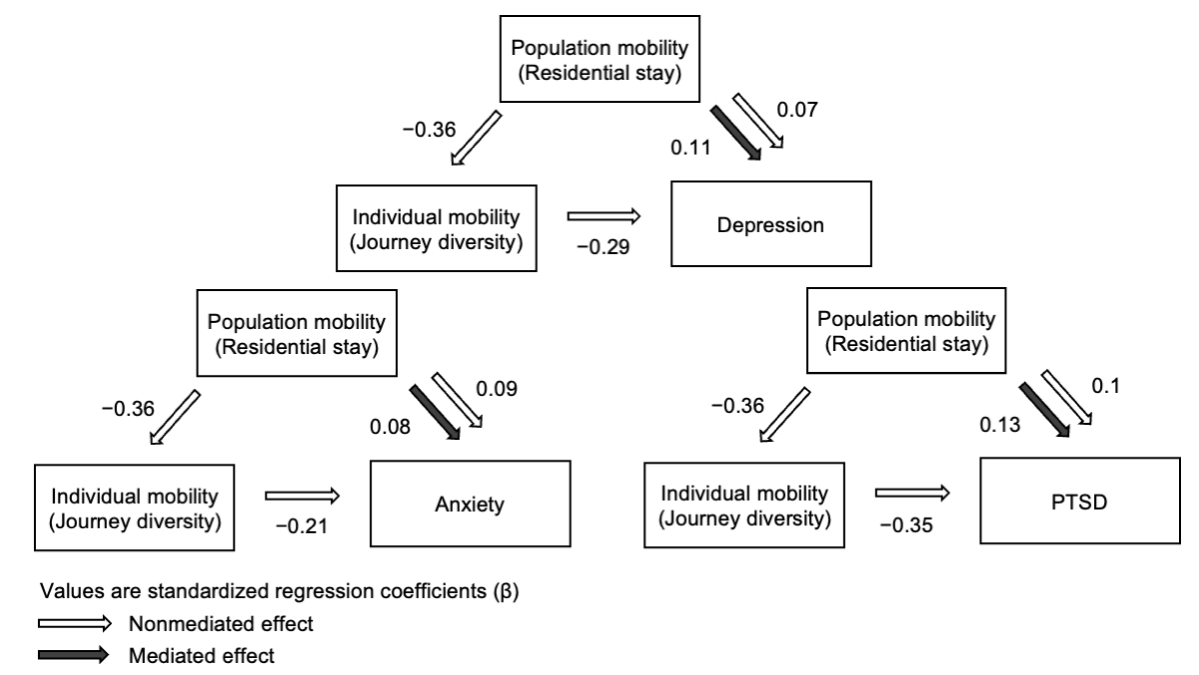
Indirect effect of population mobility on mental health via individual mobility. All effects are reported as standardized regression coefficients. Mediated effects were derived from 10,000 bootstrap samples. PTSD: posttraumatic stress disorder.

Bootstrap analyses showed indirect effects of journey diversity on the associations between population mobility and depressive symptoms (β=0.11, 95% CI 0.02-0.25) and PTSD symptoms (β=0.13, 95% CI 0.05-0.27; Figure 1; Table 2). In sensitivity analyses adjusting separately for local infection count and individual infection status, the indirect effect through journey diversity remained significant for PTSD symptoms, while the corresponding indirect effect for depression was similar in magnitude but no longer statistically significant (Multimedia Appendix 10).

Immobility and remoteness did not show significant associations with concurrent population mobility or mental health symptoms (Table 2).

**Table 2:** Concurrent associations between population mobility, individual mobility, and mental health (n=171).

Individual mobility factors and mental health symptom scales		Path A^a^			Path B^b^			Direct effect^c^			Indirect effect^d^
		β	*P* value	β		*P* value	β		*P* value	β (bootstrapped 95% CI)	
**Journey diversity**											
	PHQ-9^e^	−0.36	<.001	−0.29		.02	0.07		.64	0.11 (0.02 to 0.25)	
	GAD-7^f^	−0.36	<.001	−0.21		.07	0.09		.59	0.08 (0 to 0.2)	
	PCL-5^g^	−0.36	<.001	−0.35		.002	0.1		.48	0.13 (0.05 to 0.27)	
**Immobility**											
	PHQ-9	0.12	.14	0.19		.16	0.15		.24	0.02 (−0.01 to 0.11)	
	GAD-7	0.12	.14	0.09		.45	0.15		.30	0.01 (−0.01 to 0.07)	
	PCL-5	0.12	.14	0.09		.46	0.21		.09	0.01 (−0.01 to 0.08)	
**Remoteness**											
	PHQ-9	0.04	.54	0.04		.81	0.17		.17	0 (−0.01 to 0.03)	
	GAD-7	0.04	.54	−0.01		.97	0.17		.27	0 (−0.03 to 0.02)	
	PCL-5	0.04	.54	0.05		.70	0.22		.07	0 (−0.01 to 0.04)	

^a^Path A: association between population mobility (Hong Kong residential stay) and individual mobility.

^b^Path B: association between individual mobility and symptom level.

^c^Direct effect: association between population mobility and symptom level.

^d^Indirect effect: effect of population mobility on symptom level, mediated via individual mobility (path A×path B).

^e^PHQ-9: 9-item Patient Health Questionnaire.

^f^GAD-7: 7-item Generalized Anxiety Disorder scale.

^g^PCL-5: PTSD Checklist for DSM-5.

### Mental Health Associated With Subsequent Individual Mobility

Baseline mental health was associated with mobility patterns over time. Higher baseline depression and PTSD symptoms predicted reduced journey diversity from baseline to wave 2 (β=−0.15; *P*=.02 and β=−0.15; *P*=.01), and higher baseline PTSD symptoms predicted increased remoteness from baseline to wave 2 (β=0.19; *P*=.04; Table 3).

Additionally, changes in mental health from baseline to wave 2 were associated with mobility at wave 2, with worsening depression and PTSD symptoms associated with lower subsequent journey diversity (β=−0.32; *P*=.03 and β=−0.42; *P*=.01) and worsening anxiety associated with greater immobility (β=0.38; *P*=.01; Table 3).

**Table 3:** Individual mental health associated with subsequent individual mobility.

Time points, mental health symptom severity scales, and individual mobility factors			Mental health (time 1) predicting change in mobility (time 2−time 1)^a^		Mental health change (time 2−time 1) predicting mobility (time 2)^b^	
			β	*P* value	β	*P* value
**Time 1: wave 1; time 2: wave 2 (n=110)**						
	**PHQ-9^c^**					
		Journey diversity	−0.15	.02	−0.32	.03
		Immobility	0.07	.19	0.14	.31
		Remoteness	−0.01	.89	0.03	.87
	**GAD-7^d^**					
		Journey diversity	−0.06	.34	−0.31	.07
		Immobility	−0.01	.88	0.38	.01
		Remoteness	0.13	.20	−0.14	.45
	**PCL-5^e^**					
		Journey diversity	−0.15	.01	−0.42	.01
		Immobility	0.06	.22	0.29	.06
		Remoteness	0.19	.045	−0.03	.87
**Time 1: wave 2; time 2: wave 3 (n=49)**						
	**PHQ-9**					
		Journey diversity	0.08	.19	0	.99
		Immobility	0.03	.64	0.07	.81
		Remoteness	0.15	.13	0.54	.12
	**GAD-7**					
		Journey diversity	0.1	.11	−0.19	.62
		Immobility	0.08	.27	−0.06	.86
		Remoteness	0.07	.47	0.41	.29
	**PCL-5**					
		Journey diversity	0.07	.28	−0.28	.29
		Immobility	0.03	.68	0.22	.35
		Remoteness	0.13	.22	−0.09	.72

^a^Time 1 mobility was included as a covariate.

^b^Time 1 mental health was included as a covariate.

^c^PHQ-9: 9-item Patient Health Questionnaire.

^d^GAD-7: 7-item Generalized Anxiety Disorder scale.

^e^PCL-5: PTSD Checklist for DSM-5.

### Individual Mobility Associated With Subsequent Mental Health

Baseline mobility was associated with subsequent mental health changes. Higher baseline immobility was associated with increased anxiety symptoms from baseline to wave 2 (β=0.14; *P*=.03; Table 4).

Reduced journey diversity (baseline to wave 2) was associated with higher wave 2 depression (β=−0.43; *P*=.008) and PTSD symptoms (β=−0.41; *P*=.01); increased immobility was associated with higher subsequent anxiety (β=0.38; *P*=.03; Table 4).

**Table 4:** Individual mobility associated with subsequent individual mental health.

Time points, individual mobility factors, and mental health symptom severity scales				Mobility (time 1) predicting mental health change (time 2−time 1)^a^				Mobility change (time 2−time 1) predicting mental health (time 2)^b^		
				β		*P* value		β		*P* value
**Time 1: wave 1; time 2: wave 2 (n=110)**										
	**Journey diversity**									
		PHQ-9^c^	−0.05		.44		−0.43		.008	
		GAD-7^d^	−0.04		.41		−0.30		.06	
		PCL-5^e^	−0.05		.40		−0.41		.01	
	**Immobility**									
		PHQ-9	0.10		.18		0.18		.32	
		GAD-7	0.14		.03		0.38		.03	
		PCL-5	0.09		.16		0.32		.08	
	**Remoteness**									
		PHQ-9	−0.01		.88		0.05		.65	
		GAD-7	−0.05		.32		0		.98	
		PCL-5	−0.01		.86		−0.03		.80	
**Time 1: wave 2; time 2: wave 3 (n=49)**										
	**Journey diversity**									
		PHQ-9	−0.04		.51		0.59		.11	
		GAD-7	−0.04		.46		−0.01		.99	
		PCL-5	−0.11		.16		0.26		.42	
	**Immobility**									
		PHQ-9	0.03		.62		0.03		.93	
		GAD-7	−0.02		.81		−0.13		.66	
		PCL-5	0.14		.10		0.11		.71	
	**Remoteness**									
		PHQ-9	0.11		.08		0.29		.22	
		GAD-7	0.08		.14		0.10		.66	
		PCL-5	−0.02		.80		0.11		.59	

^a^Time 1 mental health was included as a covariate.

^b^Time 1 mobility was included as a covariate.

^c^PHQ-9: 9-item Patient Health Questionnaire.

^d^GAD-7: 7-item Generalized Anxiety Disorder scale.

^e^PCL-5: PTSD Checklist for DSM-5.

### Mediating Role of Mobility Changes in Mental Health Trajectories

The association between depressive symptoms at successive waves appeared to be partly explained by changes in journey diversity in between. Specifically, higher depression at baseline was linked to reduction in journey diversity from baseline to wave 2 (β=−0.15; *P*=.02), which was associated with higher depression at wave 2 (β=−0.43; *P*=.008). Mediation analysis found evidence for a weak indirect effect (β=0.07, 95% CI 0.002-0.19).

## Discussion

### Principal Findings

As the first study to assess pandemic-related mobility changes using both individual- and population-level smartphone data, this study examined the relationships between mobility patterns and mental health in Hong Kong. Individual geolocation data revealed 3 distinct mobility dimensions: journey diversity, immobility, and remoteness. Journey diversity demonstrated significant concurrent associations with mental health, while both journey diversity and immobility showed bidirectional longitudinal associations with mental health over time. Population-level mobility measures were not directly associated with mental health outcomes.

### Concurrent Associations Between Mobility and Mental Health

Journey diversity was significantly associated with population-level mobility shifts and individual mental health outcomes. Journey diversity—reflecting the number and variability of trips—captures selective adaptations during the pandemic, with individuals prioritizing necessary journeys (eg, groceries and work) over recreational and social travel [2]. This was corroborated by Hong Kong railway data showing reduced travel to shopping and entertainment districts [24]. Prioritizing travels by necessity, although rational, may worsen mental health, consistent with research demonstrating that variety in daily activities supports psychological well-being [25]. Neurobiologically, diverse environments may promote sensory foraging, activating underactive somatosensory processing in individuals vulnerable to depression [26]. Novel environments may shift neural activity from prefrontal to sensory integration, a pattern associated with reduced depression risk [26].

Individual-level journey diversity showed stronger and more consistent associations with mental health symptoms than population-level residential stay. Population-level residential stay showed nonsignificant total associations with PHQ-9, GAD-7, and PCL-5 symptoms, whereas journey diversity was significantly associated with PHQ-9 and PCL-5 symptoms in concurrent models. Significant individual mobility variations, even during lockdowns [27], mean population-level changes may incompletely represent individual experiences. This may explain the weak and heterogeneous findings from studies relying on lockdown status as a proxy measure [4], highlighting the need for individual-level measurements to better understand the health impacts of broader mobility shifts. Sensitivity analyses provided partial support for the robustness of these concurrent mediation findings. The indirect effect through journey diversity remained robust for PTSD symptoms after adjustment for local infection count and individual infection status. For depressive symptoms, the adjusted indirect effects were similar in magnitude to the primary estimate, but their CIs crossed 0, suggesting that the depression-related indirect effect should be interpreted as suggestive rather than robust.

### Longitudinal Bidirectional Relationships

Individual mobility and mental health were bidirectionally associated across 6-month intervals. This bidirectionality may operate through multiple mechanisms. Mental health symptoms may influence mobility through heightened perceived infection risk [8], limiting willingness to travel; depressive anhedonia may reduce anticipated pleasure from varied destinations [28]; fatigue may decrease physical capacity [29]; and executive dysfunction may impair journey planning [30]. Conversely, mobility affects mental health through exposure to varied environments [25], physical activity benefits [31], improved circadian regulation [32], and social connection opportunities [33]. Differential longitudinal relationships between mobility dimensions and symptoms (eg, journey diversity with depression and immobility with anxiety) suggest distinct yet overlapping mechanisms that warrant further investigation.

The bidirectional relationship between mobility patterns and mental health suggested a potential feedback loop. Baseline depression was associated with a decreasing journey diversity, which was associated with higher depression at wave 2 (indirect effect: β=0.07, 95% CI 0.002-0.19). These findings align with a large 17-month study where individuals with preexisting mental health conditions more likely maintained persistent confinement, which was associated with higher follow-up depressive symptoms [9]. This suggests that individuals with mental health symptoms may be more vulnerable to mobility changes during societal shifts, potentially creating a vicious cycle.

### Implications for Mobility-Based Interventions

Changes in immobility and journey diversity, but not travel remoteness, were associated with subsequent symptom level, suggesting potential targets for intervention. Maintaining diverse destinations within one’s neighborhood and reducing idle time may better support mental health than traveling long distances in a dense metropolitan area such as Hong Kong. Such targeted mobility interventions could help mitigate negative mental health impacts during restricted movement periods.

### Potential of Mobile Geolocation Data

This study contributes to the growing field of digital phenotyping by demonstrating how mobile geolocation data can reveal meaningful behavioral patterns of users [20,34-36], such as journey diversity, immobility, and remoteness. Mobile geolocation data provided standardized, objective measurements that captured mobility changes with spatial and temporal precision that would be impossible with traditional self-reports. The gain in precision and reduction in measurement error enhanced statistical power, enabling the detection of relationships that may have remained unidentified using traditional assessment methods.

Our passive data collection method minimized participant burden. Rather than requiring daily mobility logs or frequent questionnaires, participants simply downloaded GLH data from their Google accounts once, eliminating resource-intensive assessment protocols while enabling continuous monitoring.

The 3 mobility factors identified represent meaningful behavioral signatures captured in participants’ natural environments. While our study focused on pandemic-related mobility, there has been a trend of integrating multiple sensor data (eg, a mix of geolocation, screen time, app use, and microphone data) [34,37] and tracking behaviors that may signal illness progression or recovery in both mental and physical illnesses [20,34-37].

### Limitations

The sample lacked representativeness of the Hong Kong population due to the sampling frame and potential self-selection (eg, potentially excluding technology-averse or privacy-conscious individuals). Further contributing to potential bias, the analytical sample that provided baseline GLH data (n=145) differed significantly from invited CU-COVID19 participants who did not provide GLH data (n=2363), as GLH participants were older; more likely to be male; and had lower levels of education, employment, and income. While demographic weighting partially mitigated these differences, results should be interpreted considering these sample characteristics. Additionally, the predominantly mild symptom profile of participants (<13% meeting diagnostic thresholds) limits generalizability to clinical populations.

The use of Google-derived mobility data introduces additional representativeness concerns. GLH participants had compatible devices, used Google services, had location history available, and were willing to share these data. Similarly, GCMRs may better represent mobility patterns among people who use Google services and carry location-enabled devices and may underrepresent technology-averse or privacy-conscious groups.

Our analyses were constrained by statistical power issues stemming from modest sample size, sample attrition, and limited availability of GCMRs. To address these constraints, data across time points were pooled in concurrent analyses (n=171). For longitudinal analyses, temporal overlap between assessment periods necessitated separate examination of changes from wave 1 to 2 (n=110) and from wave 2 to 3 (n=49), preventing similar pooling approaches. The absence of significant associations, particularly for population-level residential stay and wave 2 to 3 longitudinal analyses, should be interpreted cautiously, as the modest sample size and repeated use of shared population-level mobility values across participants may have reduced power and precision to detect effects.

Participants began the study at different time points, introducing contextual heterogeneity; however, all variables were aligned either to the same period for concurrent analyses or to consistent intervals corresponding to follow-up survey times for longitudinal analyses. The 6-month intervals between observations did not fully leverage the continuous nature of geolocation data, leaving gaps in understanding shorter-term fluctuations in mobility–mental health relationships. Additionally, relying on 2 measurement points to infer trends across 6-month intervals makes our estimates more susceptible to random fluctuations and measurement error compared to more frequent sampling.

Despite demonstrating associations across waves, our findings remain correlational rather than causal. Sensitivity analyses adjusting for local infection count and individual infection status showed broadly similar patterns, particularly for the PTSD-related mediation finding. However, residual confounding by individual-level events, such as employment disruption, bereavement, caregiving demands, changes in work or study arrangements, or other health changes, could not be excluded. Previous quasi-experimental studies (eg, pre- or postlockdown comparisons or comparisons between regions with mobility restrictions) have aided causal inference [4], but lacked the individual-level mobility measurements available in our study. Ideally, future research could integrate both approaches using a multilevel framework that combines environmental exposure–based quasi-experimental groupings with individual-level mobility data.

The 3 GLH mobility factors resulted from data-driven factor analysis, and the names assigned (journey diversity, immobility, and remoteness) capture general relatedness among indexes, not exhaustive definitions. While the indexes counted places visited, qualitative differences between locations were not captured. Having theorized the importance of diversified sensory engagement, future research should develop measures more sensitive to place-type diversity. For population-level analysis, GCMR residential stay data were selected for consistency with individual data sources, but neither the GLH factors nor GCMRs provide comprehensive mobility measurement. GCMR residential stay was measured at the Hong Kong population level and may not capture neighborhood-level variation in local mobility conditions, built environment, social opportunities, or perceived infection risk. In addition, mobility is not equivalent to social contact. GLH and GCMRs capture movement and location patterns but not interpersonal contact, contact quality, household interaction, or perceived social support. The mental health implications of staying at home could differ by household context, although exploratory analyses did not find significant moderation by household size. GCMRs’ static baseline (January to February 2020) offers a consistent reference but fails to account for seasonality and diminishes in relevance as time progresses from the baseline period.

Finally, using Google’s proprietary technology reduced implementation and design costs but allowed limited control over data collection parameters, although external validation supports its accuracy within defined parameters [13].

### Conclusions

This study is the first to capture pandemic-related mobility changes using individualized smartphone data, revealing complex relationships between movement patterns and mental health. Individual mobility and mental health were significantly associated, though the relationships proved more nuanced than previously understood. Journey diversity showed the most consistent associations, while remoteness was least relevant. Our findings suggest a bidirectional relationship between mobility and mental health, challenging prevailing assumptions that population-level containment measures unilaterally worsen mental health outcomes.

From a public health perspective, our findings suggest that preserving safe, diverse, local mobility during public health emergencies may be relevant to mental health, although this conclusion remains tentative because the data are observational.

Smartphone data have the potential to transform health research by revealing behavioral patterns invisible to traditional methods. In this study, location tracking improved characterization of mobility–mental health relationships during the pandemic. Beyond COVID-19, the integration of movement patterns with other smartphone-captured behaviors opens new frontiers for detecting early signs of health changes and measuring intervention outcomes. As digital phenotyping matures across diverse sensor types, researchers gain increasingly nuanced insights into how everyday behaviors interact with health outcomes.

## Data Availability

The datasets used in this study are available from the corresponding author upon reasonable request. The code used for the current study is available [38].

## Appendix

Multimedia Appendix 1 Participant recruitment flowchart.

Multimedia Appendix 2 Characteristics of geolocation records.

Multimedia Appendix 3 Preprocessing of geolocation records.

Multimedia Appendix 4 Description of mobility indexes.

Multimedia Appendix 5 Factor analysis of individual mobility indexes.

Multimedia Appendix 6 Respondent vs nonrespondent characteristics.

Multimedia Appendix 7 Changes in population mobility, individual mobility, and mental health over time.

Multimedia Appendix 8 Concurrent associations between population-level residential stay and mental health symptoms.

Multimedia Appendix 9 Household members as a moderator of concurrent associations between individual mobility and mental health symptoms.

Multimedia Appendix 10 Concurrent associations between population mobility, individual mobility, and mental health after adjustment for local infection count and individual infection status.

## Abbreviations

GAD-7: 7-item Generalized Anxiety Disorder scale
GCMR: Google community mobility report
GLH: Google location history
PCL-5: posttraumatic stress disorder checklist for DSM-5
PHQ-9: 9-item Patient Health Questionnaire
PTSD: posttraumatic stress disorder
