# Supplementary material for: Mobility Patterns and Mental Health During the COVID-19 Pandemic: Longitudinal Observational Study Using Smartphone Mobility Data
Source: JMIR Form Res. 2026 Jul 27;10:e79711. doi: 10.2196/79711 (PMC13458352; doi:10.2196/79711)
Supplement: Supplementary file 2 [file formative_v10i1e79711_app10.docx]

**Concurrent associations between population mobility, individual mobility, and mental health after adjustment for local infection count and individual infection status**

| **Individual mobility** | **Mental health** | **Adjustment model** | **n** | **Path A β** | **P** | **Path B β** | **P** | **Direct effect β** | **P** | **Indirect effect β (95% CI)** |
| --- | --- | --- | --- | --- | --- | --- | --- | --- | --- | --- |
| Journey diversity | PHQ-9 | Local infection count | 171 | −0.35 | <.001 | −0.29 | .02 | 0.01 | .90 | 0.10 (-0.01, 0.32) |
| Journey diversity | PHQ-9 | Individual infection | 171 | −0.37 | <.001 | −0.30 | .01 | 0.04 | .74 | 0.11 (-0.00, 0.31) |
| Journey diversity | GAD-7 | Local infection count | 171 | −0.35 | <.001 | −0.21 | .08 | 0.03 | .80 | 0.07 (-0.02, 0.26) |
| Journey diversity | GAD-7 | Individual infection | 171 | −0.37 | <.001 | −0.22 | .06 | 0.06 | .66 | 0.08 (-0.02, 0.27) |
| Journey diversity | PCL-5 | Local infection count | 171 | −0.35 | <.001 | −0.35 | <.001 | 0.06 | .65 | 0.12 (0.03, 0.32) |
| Journey diversity | PCL-5 | Individual infection | 171 | −0.37 | <.001 | −0.36 | <.001 | 0.09 | .51 | 0.13 (0.02, 0.33) |
| Immobility | PHQ-9 | Local infection count | 171 | 0.11 | .28 | 0.19 | .18 | 0.10 | .38 | 0.02 (-0.03, 0.17) |
| Immobility | PHQ-9 | Individual infection | 171 | 0.13 | .13 | 0.20 | .14 | 0.12 | .25 | 0.03 (-0.02, 0.15) |
| Immobility | GAD-7 | Local infection count | 171 | 0.11 | .28 | 0.08 | .48 | 0.10 | .44 | 0.01 (-0.03, 0.12) |
| Immobility | GAD-7 | Individual infection | 171 | 0.13 | .13 | 0.10 | .39 | 0.13 | .31 | 0.01 (-0.03, 0.10) |
| Immobility | PCL-5 | Local infection count | 171 | 0.11 | .28 | 0.09 | .48 | 0.17 | .16 | 0.01 (-0.03, 0.13) |
| Immobility | PCL-5 | Individual infection | 171 | 0.13 | .13 | 0.10 | .44 | 0.20 | .09 | 0.01 (-0.03, 0.12) |
| Remoteness | PHQ-9 | Local infection count | 171 | −0.01 | .82 | 0.02 | .88 | 0.12 | .28 | 0.00 (-0.04, 0.03) |
| Remoteness | PHQ-9 | Individual infection | 171 | 0.03 | .69 | 0.02 | .91 | 0.15 | .17 | 0.00 (-0.03, 0.04) |
| Remoteness | GAD-7 | Local infection count | 171 | −0.01 | .82 | −0.02 | .86 | 0.11 | .40 | 0.00 (-0.03, 0.04) |
| Remoteness | GAD-7 | Individual infection | 171 | 0.03 | .69 | −0.03 | .84 | 0.14 | .27 | −0.00 (-0.05, 0.02) |
| Remoteness | PCL-5 | Local infection count | 171 | −0.01 | .82 | 0.04 | .75 | 0.18 | .13 | −0.00 (-0.05, 0.03) |
| Remoteness | PCL-5 | Individual infection | 171 | 0.03 | .69 | 0.05 | .73 | 0.22 | .07 | 0.00 (-0.02, 0.06) |

PHQ-9, Patient Health Questionnaire-9; GAD-7, General Anxiety Disorder-7; PCL-5, 4-item PTSD Checklist for DSM-5; CI, confidence interval.

Local infection count used log1p(14-day mean daily confirmed cases) over the 14 days preceding each survey. Individual infection used a wave-specific recent infection indicator, indicating infection history at wave 1, or infection in the recent 6 months in the 2^nd^ and 3^rd^ waves.

These models repeat the concurrent mediation analyses shown in Table 2 with either local infection count or individual infection status added as an additional covariate.

Path A represents the association between population mobility and individual mobility; Path B represents the association between individual mobility and mental health symptoms; the direct effect represents the association between population mobility and mental health symptoms adjusted for individual mobility; the indirect effect represents Path A x Path B. Coefficients are standardized.
