# Supplementary material for: Mobility Patterns and Mental Health During the COVID-19 Pandemic: Longitudinal Observational Study Using Smartphone Mobility Data
Source: JMIR Form Res. 2026 Jul 27;10:e79711. doi: 10.2196/79711 (PMC13458352; doi:10.2196/79711)
Supplement: Supplementary file 3 [file formative_v10i1e79711_app2.docx]

**Characteristics of geolocation records**

|  |  | Mean | SD | 25th Percentile | 75th Percentile |
| --- | --- | --- | --- | --- | --- |
| Quantity and coverage | | | | | |
|  | Coordinates per month | 9187.7 | 6522.5 | 4333.6 | 13635.7 |
|  | Place visits per month | 114.8 | 49.6 | 81.7 | 144.4 |
| Temporal and spatial sensitivity | | | | | |
|  | Record frequency (minutes between records) | 15.1 | 30 | 3.5 | 16.3 |
|  | Maximum accuracy (meters) | 184.6 | 262.1 | 71 | 195.2 |
|  |  | % |  |  |  |
| Signal source (percentage of records) | | | | | |
|  | Cellular | 10.3 |  |  |  |
|  | Wi-Fi | 64.2 |  |  |  |
|  | GPS | 6.2 |  |  |  |
|  | Others | 19.3 |  |  |  |
| Device operating system (percentage of participants) | | | | | |
|  | Android | 73.8 |  |  |  |
|  | IOS | 20 |  |  |  |
|  | Mixed | 6.2 |  |  |  |
|  |  | Median | IQR | 25th Percentile | 75th Percentile |
| Accuracy by signal source (median meters) | | | | | |
|  | Cellular | 699 | 495 | 505 | 1000 |
|  | Wi-Fi | 27 | 21 | 23 | 44 |
|  | GPS | 12 | 23 | 8 | 31 |
|  | Others | 26 | 42 | 16 | 58 |
| Accuracy by operating system (median meters) | | | | | |
|  |  | Median | IQR | 25th Percentile | 75th Percentile |
|  | Android | 26 | 10 | 21 | 31 |
|  | IOS | 35 | 10 | 27 | 37 |
|  | Mixed | 27 | 4 | 25 | 29 |
| SD, standard deviation; IQR, interquartile range | | | |  |  |
