# Supplementary material for: Mobility Patterns and Mental Health During the COVID-19 Pandemic: Longitudinal Observational Study Using Smartphone Mobility Data
Source: JMIR Form Res. 2026 Jul 27;10:e79711. doi: 10.2196/79711 (PMC13458352; doi:10.2196/79711)
Supplement: Supplementary file 5 [file formative_v10i1e79711_app4.docx]

**Description of mobility indices**

| **Name** | **Description** | **Calculation/ method** |
| --- | --- | --- |
| **Pre-processing terminology** | | |
| Transitioning | A 5-minute interval with a moving average speed > 1.5 km/h is classified as transitioning | A 5-minute interval is classified as ""transitioning"" if any of its three 10-minute moving average speeds (centered, backward, or forward) exceeded 1.5 km/h. A centered moving average (x̄ᵗᶜᵉⁿ) using data from t - 5 minutes to t + 5 minutes A backward moving average (x̄ᵗᵇᵃᶜᵏ) using data from t - 10 minutes to t A forward moving average (x̄ᵗᶠʷᵈ) using data from t to t + 10 minutes A 5-minute interval is transitioning if (x̄ᵗᶜᵉⁿ > 1.5) ∨ (x̄ᵗᵇᵃᶜᵏ > 1.5) ∨ (x̄ᵗᶠʷᵈ > 1.5) A trip is where two or more consecutive 5-minute intervals are classified as transitioning. |
| Trip | A sequence of at least two consecutive 5-minute intervals labelled as "transitioning". | - |
| Stationary block | A sequence of 5-minute intervals labelled as "stationary". | - |
| Cluster | A frequently visited area in the 14-day period. | Clusters were derived using the K-means++ algorithm, starting with K=2 and incrementing until the minimum distance between all cluster centroids exceeded 400 meters |
| Home location estimate | A cluster that most likely contain the home location at 70 days around a survey. | The home location associated with each survey was identified through a two-step process. First, location records between 2 AM and 7 AM were analyzed for the 70 days surrounding the survey date. The most frequent cluster label for each day was determined as the likely home location. Then, across the 70-day period, the cluster label most often identified as the daily home location was designated as the survey's associated home location. |
| Work location estimate | A cluster that most likely contain the work location at 70 days around a survey. | The work location associated with each survey was identified through a two-step process. First, weekday location records between 10 AM and 12 PM and between 3 PM and 5 PM were analyzed for the 70 days surrounding the survey date. The most frequent cluster label for each day was determined as the likely work location. Then, across the 70-day period, the cluster label most often identified as the daily work location was designated as the survey's associated work location. |
| Outing | A period of time between two consecutive stays at the home cluster, that may contain any number of trips or stationary periods, lasting no longer than the maximum allowed outing duration (24 hours). | To identify outings, we examine the timeline of an individual's location data. We look for periods when the person is at their home cluster, then measure the time until they next return home. If this period is 24 hours or less, it's classified as an outing. Any period away from home that exceeds 24 hours is not considered an outing. |
| Semantic Place Types | Labels describing the characteristics of a visited place that fall under 16 categories in Google Map API's documentation. | The place types for each place visit was retrieved by submitting an inquiry to https://maps.googleapis.com/maps/api/place/details/json, requesting the "type" field. |
| **Basic mobility** | | |
| Trip count | The number of distinct trips detected in the 14-day period. | Trip count = Σ(Trips) |
| Median trip duration | The median duration of all trips detected in the 14-day period in minutes. | Median Trip Duration = median(T₁, T₂, ..., Tₙ), where Tᵢ = 5 * (number of 5-minute intervals in trip i) |
| Median trip distance | The median distance of all trips detected in the 14-day period in kilometers. | For each trip j: D_j = Σ d_i, where d_i is the Euclidean distance between coordinates of consecutive 5-minute intervals in the trip Median Trip Distance = median(D₁, D₂, ..., Dₙ) Where: D_j is the total distance of trip j n is the total number of trips |
| Total stationary time | The total time spent in stationary states during the 14-day period, measured in hours. | Total Stationary Time = Σ(t_end_i - t_start_i) Where: t_start_i is the timestamp of the start of stationary block I t_end_i is the timestamp of the end of stationary block I The sum is taken over all stationary blocks in the 14-day period |
| Median outing duration | The median outing duration is calculated by determining the median duration of all outing in the 14-day period. | For each outing i: D_i = duration(outing_i) Median Outing Duration = median(D_1, D_2, ..., D_n) Where: D_i is the duration of outing i in hours n is the total number of outings |
| Median outing places | The median number of places visited during outings is calculated by determining the median of the number of unique clusters visited each outing in the 14-day period. | For each outing i: P_i = unique_clusters_visited(outing_i) Median Outing Places = median(P_1, P_2, ..., P_n) Where: P_i is the number of unique clusters visited during outing i (excluding home) unique_clusters_visited() is a function that counts distinct clusters visited n is the total number of outings |
| On-foot percentage | The percentage of 5-minute intervals where the subject was likely moving on foot during the 14-day period. | For each 5-minute interval labelled as transitioning, i: speed_i = (d_i / 5) * 60 Foot Travel Percent = (N_foot / N_total) * 100 Where: d_i is the Euclidean distance between from the coordinate s5-minutes ago speed_i is the speed at interval i in km/h N_foot = number of transitinoining 5-minute intervals with speed ≤ 5.58 km/h N_total = total number of 5-minute intervals labelled as transitioning |
| **Travel times** | | |
| Rush hour outings percent | The percentage of outings within the 14-day measurement period that begin during rush hours. Rush hours are defined as 7:30-9:30 AM, 12:30-2:00 PM, and 5:30-7:30 PM. | For each weekday outing i: R_i = 1 if start_time(outing_i) is during rush hours, 0 otherwise Rush Hour Outings Percent = (sum(R_i) / N) * 100 Where: R_i is an indicator variable: 1 if the outing starts during rush hours, 0 otherwise start_time(outing_i) is the timestamp when outing i begins N is the total number of weekday outings |
| Median home arrival time | The median time at which an individual returns home, calculated across all home arrivals during the 14-day period. The time is represented as hours past 3 AM on the same day. | Median Home Arrival Time = median(R_1, R_2, ..., R_n) Where: R_i = (arrival_hour - 3 + 24) % 24 + arrival_minute / 60.0 arrival_hour and arrival_minute are the hour and minute of each home arrival |
| Median home departure time | The median time at which an individual leaves home, calculated across all home departures during the 14-day period. The time is represented as hours past 3 AM on the same day. | Median Home Departure Time = median(D_1, D_2, ..., D_n) Where: D_i = (departure_hour - 3 + 24) % 24 + departure_minute / 60.0 departure_hour and departure_minute are the hour and minute of each home departure |
| **Home and work** | | |
| Total home time | The cumulative time spent at the identified home location during the 14-day period surrounding the survey date, measured in minutes. | Total Home Time = Σ(duration_i) where cluster_label_i = home_cluster_label Where duration_i is the duration of stationary block i in minutes. |
|  |  |  |
|  |  |  |
| Median non-home distance | The median distance from home of all stationary periods that are not at home during the 14-day period, measured in kilometers. | Median Non-Home Distance is calculated by first identifying sequences of 5-minute intervals consecutively labelled as stationary and sharing the same cluster labe. For each stationary block, the distance from home is determined based on its cluster location. Only non-home locations (distance > 0) are included. The median of these distances is then calculated.  For each stationary block i: D_i = distance_from_home(cluster_i) Median Non-Home Distance = median(D_1, D_2, ..., D_n), where D_i > 0 Where: D_i is the distance from home for stationary block I distance_from_home() is a function that returns the distance between a given cluster and home n is the total number of non-home stationary blocks |
| Total work time | The cumulative time spent at the identified work location during the 14-day period surrounding the survey date, measured in minutes. | Total Work Time = Σ(duration_i) where cluster_label_i = work_cluster_label Where duration_i is the duration of stationary block i in minutes. |
| **Places visited** | | |
| Unique places count | The number of unique clusters visited by an individual during the 14-day period. | Number of Unique Clusters = count(unique(C_1, C_2, ..., C_n)) Where: C_i = cluster label for each stationary period |
| Recreational visits | The number of place visits in the 14-day period where at least one Google Semantic Place Types is categorized under entertainment and recreation, culture, food and drink, or sports. | - |
| Errand visits | The number of place visits in the 14-day period where at least one Google Semantic Place Types is categorized under finance or services. | - |
| Transport hub visits | The number of place visits in the 14-day period where at least one Google Semantic Place Types is categorized under transportation. | - |
| Education visits | The number of place visits in the 14-day period where at least one Google Semantic Place Types is categorized under education. | - |
| Health visits | The number of place visits in the 14-day period where at least one Google Semantic Place Types is categorized under health and wellness. | - |
| Shop visits | The number of place visits in the 14-day period where at least one Google Semantic Place Types is categorized under shopping. | - |
