# Supplementary material for: Mobility Patterns and Mental Health During the COVID-19 Pandemic: Longitudinal Observational Study Using Smartphone Mobility Data
Source: JMIR Form Res. 2026 Jul 27;10:e79711. doi: 10.2196/79711 (PMC13458352; doi:10.2196/79711)
Supplement: Supplementary file 6 [file formative_v10i1e79711_app5.docx]

**Correlation matrix of 20 mobility indices at wave 1**

Seven indices that correlated at ≥0.3 with fewer than two other indices were excluded from the factor analysis: rush hour outing percentage, median home arrival time, median home departure time, errand visits, education visits, health visits and shop visits

**Scree plot of eigenvalues in exploratory factor analysis**

Both the Kaiser criterion (eigenvalues >1) and the elbow method (flattening of the curve) suggest retaining 4 factors.

| **Geomin rotated loadings from exploratory factor analysis** | | | | |
| --- | --- | --- | --- | --- |
|  | Factor | | | |
| Mobility index | 1 | 2 | 3 | 4 |
| Trips | -0.02 | -0.04 | 0.67 | -0.09 |
| Trip duration | 0.69 | 0.20 | -0.03 | 0.03 |
| Trip distance | 0.94 | 0.02 | -0.08 | -0.09 |
| On-foot % | -0.59 | 0.14 | -0.07 | 0.33 |
| Stationary time | -0.06 | 0.72 | -0.08 | 0 |
| Non-home distance | 0.61 | -0.1 | 0.19 | 0.04 |
| Home time | 0.06 | 1·01 | 0.06 | 0.05 |
| Outing duration | 0.16 | -0.55 | -0.02 | 0.45 |
| Outing places | -0.03 | -0.02 | 0.60 | 0.63 |
| Work time | 0.01 | 0.76 | -0.08 | -0.15 |
| Unique places | 0.05 | -0.02 | 0.72 | -0.05 |
| Recreation time | 0.02 | 0.02 | 0.47 | -0.26 |
| Hub time | 0.37 | 0.04 | 0.46 | 0.05 |
| An absolute loading of ≥±0.4 was used to assign indices to each factor | | | | |
| Based on the assigned mobility indices, Factors 1–3 were named "Remoteness," "Immobility," and "Journey Diversity," respectively | | | | |
| Factor 4 was excluded from confirmatory factor analysis because it included fewer than three mobility indices with loadings of ≥±0.4, the minimum required for a reliable factor structure | | | | |

| **Longitudinal confirmatory factor analysis of the journey diversity factor** | | | |
| --- | --- | --- | --- |
| Model fit | | | |
| RMSEA | 0.04 |  |  |
| CFI | 0.96 |  |  |
| TLI | 0.95 |  |  |
| SRMR | 0.09 |  |  |
| Factor loadings | | | |
|  | Standardised beta | S·E· | *P* |
| Wave 1 | | | |
| Trip count | 0.79 | 0.05 | <.001 |
| Unique places count | 0.61 | 0.07 | <.001 |
| Recreational visits | 0.47 | 0.07 | <.001 |
| Transport hub visits | 0.57 | 0.06 | <.001 |
| Median outing places | 0.42 | 0.06 | <.001 |
| Wave 2 | | | |
| Trip count | 0.9 | 0.05 | <.001 |
| Unique places count | 0.62 | 0.06 | <.001 |
| Recreational visits | 0.43 | 0.07 | <.001 |
| Transport hub visits | 0.59 | 0.06 | <.001 |
| Median outing places | 0.45 | 0.07 | <.001 |
| Wave 3 | | | |
| Trip count | 0.62 | 0.09 | <.001 |
| Unique places count | 0.56 | 0.1 | <.001 |
| Recreational visits | 0.45 | 0.08 | <.001 |
| Transport hub visits | 0.51 | 0.08 | <.001 |
| Median outing places | 0.41 | 0.08 | <.001 |
|  |  |  |  |

| **Longitudinal confirmatory factor analysis of the immobility factor** | | | |
| --- | --- | --- | --- |
| Model fit | | | |
| RMSEA | 0.06 |  |  |
| CFI | 0.97 |  |  |
| TLI | 0.96 |  |  |
| SRMR | 0.1 |  |  |
| Factor Loadings | | | |
|  | Standardised beta | S·E· | *P* |
| Wave 1 | | | |
| Total stationary time | 0.75 | 0.04 | <.001 |
| Total home time | 0.94 | 0.02 | <.001 |
| Total work time | 0.83 | 0.03 | <.001 |
| Median outing duration | -0.64 | 0.05 | <.001 |
| Wave 2 | | | |
| Total stationary time | 0.68 | 0.04 | <.001 |
| Total home time | 0.97 | 0.03 | <.001 |
| Total work time | 0.78 | 0.04 | <.001 |
| Median outing duration | -0.66 | 0.05 | <.001 |
| Wave 3 | | | |
| Total stationary time | 0.72 | 0.05 | <.001 |
| Total home time | 0.92 | 0.04 | <.001 |
| Total work time | 0.75 | 0.06 | <.001 |
| Median outing duration | -0.62 | 0.06 | <.001 |

| **Longitudinal confirmatory factor analysis of the remoteness factor** | | | |
| --- | --- | --- | --- |
| Model fit | | | |
| RMSEA | 0.08 |  |  |
| CFI | 0.94 |  |  |
| TLI | 0.92 |  |  |
| SRMR | 0.16 |  |  |
| Factor Loadings | | | |
|  | Standardised Beta | S·E· | *P* |
| Wave 1 | | | |
| Median trip duration | 0.68 | 0.04 | <.001 |
| Median trip distance | 0.99 | 0.04 | <.001 |
| Median non-home distance | 0.54 | 0.06 | <.001 |
| On-foot percentage | −0.6 | 0.05 | <.001 |
| Wave 2 | | | |
| Median trip duration | 0.6 | 0.05 | <.001 |
| Median trip distance | 1·05 | 0.04 | <.001 |
| Median non-home distance | 0.47 | 0.06 | <.001 |
| On-foot percentage | −0.65 | 0.06 | <.001 |
| Wave 3 | | | |
| Median trip duration | 0.7 | 0.06 | <.001 |
| Median trip distance | 1·05 | 0.05 | <.001 |
| Median non-home distance | 0.41 | 0.07 | <.001 |
| On-foot percentage | −0.64 | 0.07 | <.001 |
| The variable, trip distance, was excluded from the model due to issues with the residual covariance matrix (Theta), which was not positive definite. A revised model without trip distance was used for the final analysis. | | | |

| **Longitudinal confirmatory factor analysis of the remoteness factor (revised)** | | | |
| --- | --- | --- | --- |
| Model fit | | | |
| RMSEA | 0.02 |  |  |
| CFI | 1 |  |  |
| TLI | 1 |  |  |
| SRMR | 0.08 |  |  |
| Factor Loadings | | | |
|  | Standardised Beta | S·E· | *P* |
| Wave 1 | | | |
| Median trip duration | 0.5 | 0.07 | <.001 |
| Median non-home distance | 0.69 | 0.07 | <.001 |
| On-foot percentage | −0.63 | 0.08 | <.001 |
|  |  |  |  |
| Wave 2 | | | |
| Median trip duration | 0.46 | 0.07 | <.001 |
| Median non-home distance | 0.73 | 0.08 | <.001 |
| On-foot percentage | −0.77 | 0.07 | <.001 |
|  |  |  |  |
| Wave 3 | | | |
| Median trip duration | 0.57 | 0.08 | <.001 |
| Median non-home distance | 0.71 | 0.09 | <.001 |
| On-foot percentage | −0.89 | 0.08 | <.001 |
