# Supplementary material for: Mobility Patterns and Mental Health During the COVID-19 Pandemic: Longitudinal Observational Study Using Smartphone Mobility Data
Source: JMIR Form Res. 2026 Jul 27;10:e79711. doi: 10.2196/79711 (PMC13458352; doi:10.2196/79711)
Supplement: Supplementary file 7 [file formative_v10i1e79711_app6.docx]

**Respondent versus non-respondent characteristics**

|  |  | Respondents (=145) | | Non-respondents (n=2,363) | |  | | | |
| --- | --- | --- | --- | --- | --- | --- | --- | --- | --- |
| Variables | | n/ Mean | %/ SD | n/ Mean | %/ SD | Test | Statistic | df | *P* |
| Age |  | 46.9 | 15.5 | 41.3 | 13.7 | T-test (Unequal var) | −4.2 | 144 | <.001 |
| Sex | Female | 73 | 5.3 | 1540 | 65.2 | Chi-Square Test | 13.7 | 2 | .001 |
|  | Male | 72 | 49.7 | 818 | 34.6 | .. | .. | .. | .. |
|  | Others | 0 | 0 | 5 | .2 | .. | .. | .. | .. |
| Marital status | Single | 50 | 34.7 | 957 | 4.5 | Chi-Square Test | 8.5 | 4 | .07 |
|  | Married | 79 | 54.9 | 1240 | 52.5 | .. | .. | .. | .. |
|  | Divorced | 9 | 6.2 | 127 | 5.4 | .. | .. | .. | .. |
|  | Widowed | 6 | 4.2 | 33 | 1.4 | .. | .. | .. | .. |
|  | Others | 0 | 0 | 6 | .3 | .. | .. | .. | .. |
| Education | Primary of below | 13 | 9 | 62 | 2.6 | Chi-Square Test | 22.5 | 2 | <.0001 |
|  | Secondary | 58 | 40 | 818 | 34.6 | .. | .. | .. | .. |
|  | Tertiary or above | 74 | 51 | 1483 | 62.8 | .. | .. | .. | .. |
| Employment | Employed | 96 | 66.2 | 1869 | 79.3 | Chi-Square Test | 37.4 | 5 | <.0001 |
|  | Housekeeper | 5 | 3.4 | 109 | 4.6 | .. | .. | .. | .. |
|  | Others | 2 | 1.4 | 34 | 1.4 | .. | .. | .. | .. |
|  | Retired | 30 | 2.7 | 169 | 7.2 | .. | .. | .. | .. |
|  | Student | 4 | 2.8 | 98 | 4.2 | .. | .. | .. | .. |
|  | Unemployed | 8 | 5.5 | 78 | 3.3 | .. | .. | .. | .. |
| Household income (HKD) | <=13999 | 14 | 9.7 | 212 | 9 | Chi-Square Test | 12.2 | 3 | .007 |
|  | 14000-35999 | 56 | 38.6 | 626 | 26.6 | .. | .. | .. | .. |
|  | 36000-57999 | 30 | 2.7 | 498 | 21.1 | .. | .. | .. | .. |
|  | >=58000 | 45 | 31 | 1021 | 43.3 | .. | .. | .. | .. |
| Mental disorder symptoms | PHQ-9 | 4.4 | 4.7 | 4.5 | 4.7 | T-test (Equal var) | 0.4 | 2506 | .7 |
|  | GAD-7 | 3.4 | 4.1 | 3.7 | 4.3 | T-test (Equal var) | 0.7 | 2506 | .45 |
|  | PCL-5 | 2.6 | 3.1 | 2.8 | 3.2 | T-test (Equal var) | 1.1 | 2501 | .29 |
| Mental disorder^1^ | Depression | 18 | 12.4 | 319 | 13.5 | Chi-Square Test | 0.1 | 1 | .81 |
|  | Anxiety | 11 | 7.6 | 218 | 9.2 | Chi-Square Test | 0.3 | 1 | .61 |
|  | PTSD | 18 | 12.4 | 350 | 14.8 | Chi-Square Test | 0.5 | 1 | .5 |
| Past mental disorder | Depression | 12 | 8.3 | 133 | 5.6 | Chi-Square Test | 1.4 | 1 | .24 |
|  | Anxiety | 23 | 16 | 413 | 17.5 | Chi-Square Test | 0.1 | 1 | .73 |
|  | Any Mental Disorder^2^ | 35 | 24.3 | 531 | 22.5 | Chi-Square Test | 0.2 | 1 | .68 |
| Mental health treatment | Lifetime | 18 | 12.5 | 229 | 9.7 | Chi-Square Test | 0.9 | 1 | .35 |
| SD, standard deviation, df; degrees of freedom; PHQ-9, Patient Health Questionnaire-9; GAD-7, General Anxiety Disorder-7; PCL-5, 4-item PTSD Checklist for DSM-5 | | | | | | | | | |
| 1 Positive screenings for depression, anxiety, and PTSD were defined as scores of 10 or greater on the PHQ-9 and GAD-7, and a score of 7 or greater on the PCL-5, respectively. | | | | | | | | | |
| 2 Any past disorder/symptom from depression, mania or bipolar disorder, panic attack or panic disorder, anxiety-related problem, problems with alcohol consumption, abuse or dependence or problems with drug use | | | | | | | | | |
