# Supplementary material for: Mobility Patterns and Mental Health During the COVID-19 Pandemic: Longitudinal Observational Study Using Smartphone Mobility Data
Source: JMIR Form Res. 2026 Jul 27;10:e79711. doi: 10.2196/79711 (PMC13458352; doi:10.2196/79711)
Supplement: Supplementary file 8 [file formative_v10i1e79711_app7.docx]

**Changes in population mobility, individual mobility and mental health over time**

|  | Wave 1 | | Wave 2 | | Wave 3 | | Change over time | | |  |
| --- | --- | --- | --- | --- | --- | --- | --- | --- | --- | --- |
|  |  |  |  |  |  |  |  |  |  |  |
|  | Mean | SD | Mean | SD | Mean | SD | Statistic | df | *P* |  |
| **Population mobility** | |  |  |  |  |  |  |  |  |  |
| GCMR residential stay | 7.9 | 3.5 | 7 | 4.1 | 6.9 | 0.1 | 3 | 2 | .22 |  |
| **Individual mobility** | |  |  |  |  |  |  |  |  |  |
| Journey diversity | 34.8 | 15.8 | 4.8 | 18.6 | 38.2 | 13.9 | 19.3 | 2 | <.001 |  |
| Immobility | 12.5 | 3.5 | 12.9 | 3.3 | 13 | 2.7 | 1.6 | 2 | .44 |  |
| Remoteness | 28 | 2.6 | 28.3 | 3.5 | 28.6 | 3.8 | 12.6 | 2 | .002 |  |
| **Mental health** | |  |  |  |  |  |  |  |  |  |
| PHQ-9 | 4.4 | 4.7 | 4.9 | 4.7 | 4.2 | 4 | 1.8 | 2 | .42 |  |
| GAD-7 | 3.4 | 4.1 | 3.6 | 3.8 | 3.3 | 3.8 | 0 | 2 | 1 |  |
| PCL-5 | 2.6 | 3.1 | 2.8 | 3.1 | 2.6 | 3.1 | 0.5 | 2 | .76 |  |
| SD, Standard deviation; GCMR, Google Community Mobility Reports; PHQ-9, Patient Health Questionnaire-9; GAD-7, General Anxiety Disorder-7; PCL-5, 4-item PTSD Checklist for DSM-5; df; degrees of freedom | | | | | | | | | |  |
