# Supplementary material for: Mobility Patterns and Mental Health During the COVID-19 Pandemic: Longitudinal Observational Study Using Smartphone Mobility Data
Source: JMIR Form Res. 2026 Jul 27;10:e79711. doi: 10.2196/79711 (PMC13458352; doi:10.2196/79711)
Supplement: Supplementary file 9 [file formative_v10i1e79711_app8.docx]

**Concurrent associations between population-level residential stay and mental health symptoms**

| **Mental health outcome** | **n** | **β** | **95% CI** | **P** |
| --- | --- | --- | --- | --- |
| PHQ-9 | 171 | 0.17 | (−0.15, 0.42) | .172 |
| GAD-7 | 171 | 0.16 | (−0.18, 0.45) | .269 |
| PCL-5 | 171 | 0.22 | (−0.10, 0.50) | .069 |

PHQ-9, Patient Health Questionnaire-9; GAD-7, General Anxiety Disorder-7; PCL-5, 4-item PTSD Checklist for DSM-5; CI, confidence interval.

The estimates in this table are total associations, estimated without individual mobility factors in the model. They differ from the Table 2 direct effects, which adjust for the corresponding individual mobility factor in each mediation model.

Population-level mobility was measured using Google Community Mobility Reports residential stay over the 14 days preceding each survey. Coefficients are standardized.
