# Supplementary material for: Mobility Patterns and Mental Health During the COVID-19 Pandemic: Longitudinal Observational Study Using Smartphone Mobility Data
Source: JMIR Form Res. 2026 Jul 27;10:e79711. doi: 10.2196/79711 (PMC13458352; doi:10.2196/79711)
Supplement: Supplementary file 10 [file formative_v10i1e79711_app9.docx]

**Household members as a moderator of concurrent associations between individual mobility and mental health symptoms**

| **Mobility factor** | **Outcome** | **n** | **Original mobility β** | **Original P** | **Mobility β in moderation model** | **P** | **Mobility x household members β** | **Interaction P** |
| --- | --- | --- | --- | --- | --- | --- | --- | --- |
| Journey diversity | PHQ-9 | 171 | −0.29 | .02 | −0.37 | .003 | 0.13 | .34 |
| Journey diversity | GAD-7 | 171 | −0.21 | .07 | −0.26 | .03 | 0.14 | .29 |
| Journey diversity | PCL-5 | 171 | −0.35 | <.001 | −0.41 | <.001 | 0.17 | .12 |
| Immobility | PHQ-9 | 171 | 0.19 | .16 | 0.24 | .07 | −0.05 | .56 |
| Immobility | GAD-7 | 171 | 0.09 | .45 | 0.12 | .32 | −0.05 | .48 |
| Immobility | PCL-5 | 171 | 0.09 | .46 | 0.10 | .45 | 0.00 | .95 |
| Remoteness | PHQ-9 | 171 | 0.04 | .81 | 0.01 | .96 | 0.07 | .66 |
| Remoteness | GAD-7 | 171 | −0.01 | .96 | −0.03 | .82 | 0.07 | .59 |
| Remoteness | PCL-5 | 171 | 0.05 | .70 | 0.05 | .71 | 0.03 | .84 |

PHQ-9, Patient Health Questionnaire-9; GAD-7, General Anxiety Disorder-7; PCL-5, 4-item PTSD Checklist for DSM-5.

The original mobility–symptom β refers to the association between each individual mobility factor and each mental health symptom score (Table 2, Path B), before adding the household-members interaction term.

Coefficients are standardized.
