# Supplementary material for: Mobility Patterns and Mental Health During the COVID-19 Pandemic: Longitudinal Observational Study Using Smartphone Mobility Data
Source: JMIR Form Res. 2026 Jul 27;10:e79711. doi: 10.2196/79711 (PMC13458352; doi:10.2196/79711)
Supplement: Supplementary file 12 [file formative_v10i1e79711_app3.docx]

**Preprocessing of geolocation records**
